# Association of professional environment with loneliness and perceived social isolation among individuals in their second half of life

**DOI:** 10.1007/s40520-025-03095-4

**Published:** 2025-06-23

**Authors:** André Hajek, Razak M. Gyasi, Dong Keon Yon, Supa Pengpid, Karl Peltzer, Hans-Helmut König

**Affiliations:** 1 Department of Health Economics and Health Services Research, University Medical Center Hamburg-Eppendorf, Hamburg Center for Health Economics, Martinistraße 52, 20246 Hamburg, Germany; 2 African Population and Health Research Center, Nairobi, Kenya; 3 National Centre for Naturopathic Medicine, Faculty of Health, Southern Cross University, Lismore, NSW Australia; 4 Center for Digital Health, Medical Science Research Institute, Kyung Hee University College of Medicine, Seoul, South Korea; 5 Department of Health Education and Behavioral Sciences, Faculty of Public Health, Mahidol University, Bangkok, Thailand; 6 Department of Public Health, Sefako Makgatho Health Sciences University, Pretoria, South Africa; 7 Department of Healthcare Administration, College of Medical and Health Science, Asia University, Taichung, Taiwan; 8 Department of Psychology, University of the Free State, Bloemfontein, South Africa; 9 Department of Psychology, College of Medical and Health Science, Asia University, Taichung, Taiwan

**Keywords:** Loneliness, Social isolation, Professional environment, Sex differences, Age differences, German Ageing Survey

## Abstract

**Background:**

Little attention has been paid to the association of job-related factors with loneliness and perceived social isolation. More specifically, studies are missing examining how professional environment is associated with loneliness and perceived social isolation.

**Aims:**

To examine how the professional environment is associated with loneliness and perceived social isolation (also stratified by sex and age group).

**Methods:**

Data were taken from the latest wave of the nationally representative German Ageing Survey (focusing on individuals aged 43 and older living in private households, *n* = 3,576, mean age: 68.6 years, SD: 11.1 years, with 52.1% being female). Occupations were grouped into six professional environments (realistic, investigative, artistic, social, enterprising, and conventional professions) grounded on Holland’s model. More than 550 occupations were classified to one of those six categories based on Stuth’s approach. Loneliness and perceived social isolation were quantified using established tools.

**Results:**

Our findings revealed that individuals in social (β=-0.08, *p* < 0.01), enterprising (β=-0.09, *p* < 0.01), and conventional professions (β=-0.07, *p* < 0.05) had lower perceived social isolation scores compared to those in realistic professions among the total sample (and particularly among those aged 65 and older); however, no differences in loneliness were observed. Notably, some effects were sex-specific, with men in conventional professions and women in artistic professions experiencing lower perceived social isolation scores. Enterprising professions in particular mainly yielded positive outcomes across groups.

**Discussion:**

Our findings underlined the association between professional environment and perceived social isolation, varying by age and sex.

**Conclusions:**

Enterprising professions in particular may assist in avoiding perceived social isolation, pending longitudinal evidence. Studies from other countries are recommended.

**Supplementary Information:**

The online version contains supplementary material available at 10.1007/s40520-025-03095-4.

## Introduction

It is generally projected that the number of individuals in old age will substantially increase in the next decades. In later life, various events can take place such as spousal loss, losses of functional or cognitive abilities, which can ultimately contribute to loneliness (subjective discrepancy between actual and desired social relationships [1]; see also: [2, 3]) or perceived social isolation (feeling that one does not belong to the society [4]). Loneliness and social isolation in turn are associated with chronic illnesses and mortality [5, 6].

A bulk of studies have identified factors associated with loneliness and perceived social isolation. For example, it has been shown that being married or voluntary activities are associated with lower loneliness and social isolation scores [7–9]. Other research showed that self-rated health is associated with favourable loneliness and isolation levels [10, 11].

Much lower attention has been paid to the association of job-related factors with loneliness and perceived social isolation. For example, some studies have shown that unemployment is associated with higher loneliness or social isolation levels [12]. Other research has demonstrated that higher occupational prestige is associated with lower loneliness [13].

Studies are missing examining how professional environment is associated with loneliness and perceived social isolation. Thus, our aim was to fill this gap by investigating how the professional environment is associated with loneliness and perceived social isolation (also stratified by sex and age group). This can considerably enrich our current knowledge in this area identifying possible side effects (in terms of psychosocial consequences) of the professional environment.

The underlying idea is that specific professions have certain environments with typical activities. Such professions can be classified according to the RIASEC (acronym which stands for: Realistic, Investigative, Artistic, Social, Enterprising, and Conventional; details are displayed in the methods section) model proposed by Holland [14]. While, for example, investigative professions may often involve a lot of thinking, observing and analysing, other professional environments such as enterprising professions involve a lot of work with people and social professions involve curing, helping, or training.

An association of loneliness or perceived social isolation with professional environment seems conceivable for a variety of reasons. Individuals may develop an identity through their professional environment (with its specific characteristics and usual activities). For example, if individuals go into entrepreneurial professions, and increasingly see themselves as entrepreneurs over the years, this can also have an impact on their sociability [15]. More generally, the professional environment could shape our contacts and social relationships in general. More precisely, it may be that social relationships have developed in and outside of the professional environment which can help prevent loneliness and perceived social isolation, even after the active professional career has ended. Additionally, the way in which society values and judges such professional environments may be associated with self-esteem which in turn may drive loneliness or perceived social isolation.

## Methods

### Sample

Data were obtained from wave 8 of the German Ageing Survey (DEAS), with data collection occurring between December 2022 and mid-2023. Participants were interviewed both via telephone (CATI) and in person (CAPI). The response rate was about 62%. Each interview lasted about 81 min. Upon completing the interview, participants had the option to fill out an additional questionnaire (by post or online; mostly including more sensitive topics such as satisfaction with life or perceived social isolation).

Informed consent was obtained from all individuals participating in the DEAS study. The DEAS study was conducted following the Declaration of Helsinki and its later amendments. An ethical review was deemed unnecessary, as the study did not fulfill the criteria for such an ethical review. For instance, it did not involve patient examinations or present risks to participants.

### Outcomes: loneliness and perceived social isolation

The well-known De Jong Gierveld scale [16] was used to measure loneliness. Three items were recoded. By averaging all items, a total score was calculated. The score ranges from 1 to 4, with higher scores indicating greater levels of loneliness. The reliability of the scale was substantiated in this study (Cronbach’s alpha and McDonald’s omega of 0.84).

The four-item Bude and Lantermann instrument [4] was used to assess perceived social isolation. The total score was calculated by averaging responses across all four items, with scores varying from 1 to 4, whereby higher scores indicate greater perceived social isolation. The reliability of this measure was also confirmed (Cronbach’s alpha and McDonald’s omega of 0.87).

### Independent variables of interest: professional environment

Occupations were grouped into six professional environments. Such environments are grounded on Holland [14]. The underlying assumption is that professions can be characterized as specific work environments associated with particular activities that are typical of such occupations. Main activities carried out by individuals in their professions were identified using data from more than 280,000 employed individuals in the German microcensus 2018. Overall, 554 occupations were classified to one of six categories [17]: Realistic professions (encompassing hands-on roles, in crafts in particular), investigative professions (mainly referring to research-oriented professions), artistic professions (centered on creative fields), social professions (including nursing, caregiving or teaching roles), enterprising professions (for example, those oriented towards advertising, management or sales), or conventional professions (e.g., managing and executing predominantly structured tasks within organizational hierarchies, such as administrative functions) [18]. Additional details are provided by Stuth [17, 19]. For individuals who had already retired, the last job is used as the basis for the classification.

### Covariates

Guided by former research [20–24], several covariates were included in regression analysis. Age, sex (men; women), relationship status (married and cohabiting with spouse; married but not living together; single; widowed; divorced), job status (currently employed, retired, and other), level of educational attainment (ISCED framework [25]: low, medium, and high education), and monthly household net income (in Euro) were used as socio-economic factors.

With regard to lifestyle factors, the frequency of physical activity (categories: daily, several times a week, once a week, 1–3 times a month, less often, and never) was used. Additionally, alcohol intake was assessed using the same response categories (from never to daily) and smoking status was also included in regression analysis as covariate (yes, daily; yes, occasionally; no, not anymore; never been a smoker).

Regarding health-related factors, probable depression, physical functioning and the existence of chronic illnesses were added as covariates to our regression model. Probable depression was assessed based on the 15-item version of the Center for Epidemiologic Studies Depression Scale [26], producing a total score varying from 0 to 45, whereby higher values reflect more severe depressive symptoms. Individuals were deemed to have probable depression if their total score was 18 or above. Physical functioning was measured via the physical functioning subscale of the SF-36 [27], resulting in a total from 0 to 100, with higher scores corresponding to better physical abilities. Additionally, a cumulative score of chronic physical illnesses was determined based on the absence (0) or presence (1) of eleven chronic conditions (e.g., heart and circulatory disorders, diabetes, or cancer).

### Statistical analysis

The sample characteristics are presented first (also stratified by professional environment), followed by linear regression analyses (with robust standard errors). As outcome measures, we used loneliness and perceived social isolation. Findings were also stratified by sex and age group (43 to 64 years - mainly referring to the i.e. the usual working age; 65 years and over). The professional environment served as key independent variable. It was adjusted for a wide array of covariates listed in the section “Covariates”.

McDonald’s omega was computed using a tool created by Shaw [28]. Statistical significance was defined as a p-value of less than 0.05. Marginal significance was defined as a p-value between 0.05 and 0.10. All analyses were conducted with StataNow MP 18.5 (Stata Corp., College Station, Texas).

## Results

### Sample characteristics

In Table 1, the characteristics of the analytic sample (*n* = 3,576; total sample and also stratified by professional environment) are displayed. It should be noted that the analytic sample (*n* = 3,576 individuals) described here refers to the model in which loneliness served as the outcome variable. The model with social isolation as the outcome differs by one individual (*n* = 3,577). Due to this very small discrepancy in sample size, the analytical sample with social isolation as the outcome was not described separately.

In the total sample, mean age was 68.6 years (SD: 11.1 years; 43 to 99 years) and 52.1% of the participants were female. Mean loneliness score was 1.7 (SD: 0.5) and mean perceived social isolation score was 1.6 (SD: 0.6) in the total sample. Depending on the professional environment, mean loneliness scores varied between 1.7 (e.g., individuals with enterprising profession: 1.7, SD: 0.5) and 1.8 (e.g., individuals realistic profession: 1.8, SD: 0.5). Mean perceived social isolation scores varied from 1.5 (SD: 0.7, individuals with artistic profession) and 1.7 (SD: 0.6, individuals with realistic profession). Further details are presented in Table 1.

**Table 1:** Characteristics of the sample (*n* = 3,576 individuals, wave 8)

	Realistic	Investigative	Artistic	Social	Enterprising	Conventional	Total	*P*-value
n (%)	750 (21.0)	250 (7.0)	27 (0.8)	997 (27.9)	688 (19.2)	864 (24.2)	3576 (100.0)	
Age: Mean (SD)	69.1 (10.6)	68.0 (12.3)	67.0 (11.4)	67.3 (11.0)	69.6 (11.4)	69.2 (11.0)	68.6 (11.1)	< 0.001
Sex: N (%)								< 0.001
Men	548 (73.1)	175 (70.0)	10 (37.0)	295 (29.6)	377 (54.8)	307 (35.5)	1712 (47.9)	
Women	202 (26.9)	75 (30.0)	17 (63.0)	702 (70.4)	311 (45.2)	557 (64.5)	1864 (52.1)	
Marital status: N (%)								0.09
Married, living together with spouse	494 (65.9)	185 (74.0)	15 (55.6)	649 (65.1)	475 (69.0)	579 (67.0)	2397 (67.0)	
Married, living separated from spouse	6 (0.8)	1 (0.4)	1 (3.7)	15 (1.5)	8 (1.2)	6 (0.7)	37 (1.0)	
Divorced	73 (9.7)	21 (8.4)	4 (14.8)	107 (10.7)	61 (8.9)	90 (10.4)	356 (10.0)	
Widowed	112 (14.9)	24 (9.6)	4 (14.8)	138 (13.8)	102 (14.8)	140 (16.2)	520 (14.5)	
Single	65 (8.7)	19 (7.6)	3 (11.1)	88 (8.8)	42 (6.1)	49 (5.7)	266 (7.4)	
Employment status: N (%)								0.08
Employed	194 (25.9)	81 (32.4)	9 (33.3)	320 (32.1)	188 (27.3)	235 (27.2)	1027 (28.7)	
Retired	518 (69.1)	158 (63.2)	16 (59.3)	626 (62.8)	468 (68.0)	598 (69.2)	2384 (66.7)	
Other: not employed	38 (5.1)	11 (4.4)	2 (7.4)	51 (5.1)	32 (4.7)	31 (3.6)	165 (4.6)	
Education: N (%)								< 0.001
Low	43 (5.7)	2 (0.8)	1 (3.7)	16 (1.6)	23 (3.3)	23 (2.7)	108 (3.0)	
Medium	505 (67.3)	36 (14.4)	5 (18.5)	290 (29.1)	322 (46.8)	507 (58.7)	1665 (46.6)	
High	202 (26.9)	212 (84.8)	21 (77.8)	691 (69.3)	343 (49.9)	334 (38.7)	1803 (50.4)	
Household net income: Mean (SD)	2795.9 (1498.4)	4305.8 (2176.3)	3400.0 (1634.0)	3856.5 (2130.0)	4089.8 (3574.1)	3686.8 (2750.2)	3665.9 (2568.5)	< 0.001
Smoking behavior: N (%)								<0.01
Yes, daily	113 (15.1)	19 (7.6)	1 (3.7)	99 (9.9)	67 (9.7)	80 (9.3)	379 (10.6)	
Yes, occassionally	33 (4.4)	12 (4.8)	0 (0.0)	35 (3.5)	29 (4.2)	27 (3.1)	136 (3.8)	
No, not anymore	299 (39.9)	97 (38.8)	11 (40.7)	360 (36.1)	278 (40.4)	345 (39.9)	1390 (38.9)	
Never been a smoker	305 (40.7)	122 (48.8)	15 (55.6)	503 (50.5)	314 (45.6)	412 (47.7)	1671 (46.7)	
Sports activity (frequency): N (%)								< 0.001
Daily	48 (6.4)	19 (7.6)	5 (18.5)	123 (12.3)	79 (11.5)	92 (10.6)	366 (10.2)	
Several times a week	158 (21.1)	101 (40.4)	10 (37.0)	352 (35.3)	235 (34.2)	290 (33.6)	1146 (32.0)	
Once a week	118 (15.7)	43 (17.2)	5 (18.5)	183 (18.4)	109 (15.8)	157 (18.2)	615 (17.2)	
1–3 times a month	45 (6.0)	17 (6.8)	1 (3.7)	55 (5.5)	39 (5.7)	50 (5.8)	207 (5.8)	
Less often	100 (13.3)	16 (6.4)	1 (3.7)	115 (11.5)	62 (9.0)	70 (8.1)	364 (10.2)	
Never	281 (37.5)	54 (21.6)	5 (18.5)	169 (17.0)	164 (23.8)	205 (23.7)	878 (24.6)	
Alcohol consumption (frequency): N (%)								< 0.001
Daily	89 (11.9)	31 (12.4)	2 (7.4)	99 (9.9)	76 (11.0)	83 (9.6)	380 (10.6)	
Several times a week	172 (22.9)	90 (36.0)	4 (14.8)	249 (25.0)	200 (29.1)	213 (24.7)	928 (26.0)	
Once a week	115 (15.3)	51 (20.4)	4 (14.8)	180 (18.1)	122 (17.7)	117 (13.5)	589 (16.5)	
1–3 times a month	82 (10.9)	25 (10.0)	8 (29.6)	147 (14.7)	94 (13.7)	126 (14.6)	482 (13.5)	
Less often	199 (26.5)	36 (14.4)	8 (29.6)	221 (22.2)	140 (20.3)	228 (26.4)	832 (23.3)	
Never	93 (12.4)	17 (6.8)	1 (3.7)	101 (10.1)	56 (8.1)	97 (11.2)	365 (10.2)	
Number of chronic illnesses (from 0 to 11): Mean (SD)	3.0 (2.1)	2.4 (1.8)	2.3 (1.7)	2.6 (1.9)	2.7 (2.1)	2.7 (2.0)	2.7 (2.0)	< 0.001
Physical functioning (Physical functioning subscale of the SF-36, from 0 to 100; with higher scores corresponding to better physical functioning): Mean (SD)	78.6 (24.0)	84.8 (22.1)	80.9 (29.0)	83.8 (20.4)	82.2 (21.8)	80.3 (23.5)	81.6 (22.5)	< 0.001
Probable depression: N (%)								
Absence	696 (92.8)	236 (94.4)	27 (100.0)	939 (94.2)	652 (94.8)	801 (92.7)	3351 (93.7)	
Presence	54 (7.2)	14 (5.6)	0 (0.0)	58 (5.8)	36 (5.2)	63 (7.3)	225 (6.3)	0.29
Loneliness (DJG tool, 1 to 4, with higher values corresponding to higher loneliness levels)	1.8 (0.5)	1.7 (0.6)	1.7 (0.6)	1.7 (0.6)	1.7 (0.5)	1.8 (0.5)	1.7 (0.5)	< 0.001
Perceived social isolation (Bude/Lantermann tool, 1 to 4, with higher values corresponding to higher perceived social isolation levels)	1.7 (0.6)	1.6 (0.6)	1.5 (0.7)	1.6 (0.6)	1.6 (0.6)	1.6 (0.6)	1.6 (0.6)	< 0.001

### Regression analysis

Adjusted linear regressions (Table 2) showed that individuals with social (β=-0.08, *p* < 0.01), enterprising (β=-0.09, *p* < 0.01), and conventional professions (β=-0.07, *p* < 0.05) had significantly lower perceived social isolation scores compared to individuals with realistic professions among the total sample. Worth noting that there was a marginal significant perceived social isolation difference between individuals with investigative profession compared to individuals with realistic professions (β=-0.07, *p* < 0.10). No significant associations between the different groups were identified when loneliness served as outcome measure.

**Table 2:** Association of professional environment with loneliness and perceived social isolation among the total sample. Results of linear regressions (wave 8)

	Loneliness	Perceived social isolation
Investigative profession (Reference category: Realistic profession)	-0.02	-0.07+
	(-0.10–0.06)	(-0.15–0.00)
Artistic profession	-0.04	-0.07
	(-0.25–0.17)	(-0.31–0.18)
Social profession	-0.04	-0.08**
	(-0.10–0.01)	(-0.14 - -0.02)
Enterprising profession	-0.04	-0.09**
	(-0.09–0.02)	(-0.15 - -0.04)
Conventional profession	0.01	-0.07*
	(-0.05–0.06)	(-0.13 - -0.01)
Covariates	✓	✓
Individuals	3,576	3,577
R²	0.11	0.15

Comments: Unstandardized beta coefficients are presented. 95% CI in parentheses; *** *p* < 0.001, ** *p* < 0.01, * *p* < 0.05, + *p* < 0.10. Covariates include: age, sex, marital status, education, employment status, household net income, smoking behaviour, frequency of sports activity, alcohol consumption, number of chronic conditions, physical functioning, and probable depression. The sample sizes slightly varied because of the different numbers of missings in the outcomes used. Listwise deletion was used to handle missings

Stratified by sex (Table 3), individuals with artistic (β=-0.27, *p* < 0.05) and enterprising professions (β=-0.10, *p* < 0.05) had significantly lower perceived social isolation scores compared to individuals with realistic professions among women. Moreover, individuals with conventional (β=-0.14, *p* < 0.001) had significantly lower perceived social isolation scores compared to individuals with realistic professions among men. There were no significant loneliness differences between the professional environments in both sexes.

Stratified by age group (Table 3), individuals with enterprising professions had lower loneliness scores (β=-0.10, *p* < 0.05) compared to individuals with realistic professions among individuals aged 43 to 64 years. No loneliness differences were observed among individuals aged 65 years and over.

Among individuals aged 43 years to 64 years, there were no significant differences in perceived social isolation according to professional environment. In contrast, among individuals aged 65 years and over, individuals with social (β=-0.11, *p* < 0.01), enterprising (β=-0.12, *p* < 0.001), and conventional professions (β=-0.09, *p* < 0.05) had significantly lower perceived social isolation scores compared to individuals with realistic professions.

It is worth noting that we did a robustness check where we additionally adjusted for wealth (own or spouse’s assets). However, findings remained virtually the same. We also performed an additional analysis where we only included individuals currently employed. Such findings were very similar compared to the results for the individuals aged 43 to 64 years. In a further robustness check, ordered probit regressions were estimated (rather than linear regressions). However, findings remained virtually the same (in terms of significance). Of note also that there were also significant differences between group 1 and the aforementioned groups in the total sample (there were no significant differences between the other groups; please see the Appendix).

**Table 3:** Association of professional environment with loneliness and perceived social isolation stratified by sex and age group. Results of linear regressions (wave 8)

	Loneliness among men	Loneliness among women	Perceived social isolation among men	Perceived social isolation among women	Loneliness among individuals aged 43 to 64 years	Loneliness among individuals aged 65 years and over	Perceived social isolation among individuals aged 43 to 64 years	Perceived social isolation among individuals aged 65 years and over
Investigative profession (Reference category: Realistic profession)	-0.00	-0.07	-0.05	-0.11	-0.05	-0.01	-0.06	-0.08
	(-0.10–0.09)	(-0.22–0.07)	(-0.15–0.05)	(-0.25–0.03)	(-0.19–0.08)	(-0.10–0.09)	(-0.19–0.07)	(-0.18–0.02)
Artistic profession	0.12	-0.16	0.26	-0.27*	-0.19	0.04	-0.16	-0.01
	(-0.25–0.49)	(-0.41–0.09)	(-0.20–0.71)	(-0.50 - -0.04)	(-0.52–0.15)	(-0.23–0.31)	(-0.47–0.14)	(-0.36–0.33)
Social profession	-0.01	-0.08+	-0.07+	-0.07	-0.08	-0.03	-0.04	-0.11**
	(-0.09–0.06)	(-0.17–0.01)	(-0.15–0.01)	(-0.16–0.02)	(-0.17–0.02)	(-0.10–0.04)	(-0.14–0.05)	(-0.19 - -0.04)
Enterprising profession	-0.02	-0.07	-0.07+	-0.10*	-0.10*	-0.02	-0.05	-0.12***
	(-0.09–0.05)	(-0.16–0.02)	(-0.14–0.01)	(-0.20 - -0.00)	(-0.20 - -0.00)	(-0.08–0.05)	(-0.15–0.05)	(-0.19 - -0.05)
Conventional profession	-0.01	-0.01	-0.14***	-0.01	-0.00	0.01	-0.03	-0.09*
	(-0.08–0.06)	(-0.09–0.08)	(-0.21 - -0.06)	(-0.10–0.08)	(-0.10–0.09)	(-0.06–0.07)	(-0.13–0.06)	(-0.16 - -0.02)
Covariates	✓	✓	✓	✓	✓	✓	✓	✓
Individuals	1,712	1,864	1,714	1,863	1,208	2,368	1,211	2,366
R²	0.12	0.09	0.20	0.13	0.14	0.10	0.19	0.14

Comments: Unstandardized beta coefficients are presented. 95% CI in parentheses; *** *p* < 0.001, ** *p* < 0.01, * *p* < 0.05, + *p* < 0.10. Covariates include: age, sex (if appropriate), marital status, education, employment status, household net income, smoking behaviour, frequency of sports activity, alcohol consumption, number of chronic conditions, physical functioning, and probable depression. Listwise deletion was used to handle missings

## Discussion

Based on data from a large sample representative for community-dwelling individuals in their second half of life, the objective of this study was to investigate how professional environment is associated with loneliness and perceived social isolation. We found that– compared to individuals with realistic professions– individuals with social, enterprising, and conventional professions had lower perceived social isolation scores (particularly among those aged 65 and over, who are often already retired), whilst no loneliness differences were identified. Some associations were sex-specific. For example, compared to individuals with realistic professions, individuals with conventional professions had lower perceived social isolation scores solely among men, whereas individuals with artistic professions had lower perceived social isolation scores solely among women. Particularly enterprising professions were associated with favorable outcomes across the groups. This study makes a significant contribution to our current knowledge in this research area by being the first to examine the relationship of professional environment with loneliness and perceived social isolation.

The existing studies mainly focused on the employment situation more broadly suggesting that individuals being employed showed lower loneliness scores than individuals being unemployed [29]. One study showed that temporary workers had higher loneliness at work levels compared to individuals with permanent contracts in Flanders, Belgium [30]. A former German study demonstrated a link between higher professional prestige and lower loneliness levels [13].

It is of note that individuals with realistic professions often have mechanical abilities and tend to work with animals, plants, machines or objects (such as electricians, mechanics, carpenters or woodworkers). Despite the often physical nature of the work, they may sometimes feel that they do not belong to certain sections of society or may feel undervalued by others (even after retirement). Such reactions by others can decrease self-worth, ultimately leading to higher perceived social isolation levels. This may explain our current findings. In contrast, individuals with social professions such as nurses may be intrinsically motivated for their job, enjoy to work with people, for example to help them [31]. Such individuals could see their work as particularly meaningful and valued by society and the individuals they serve. In a similar vein, a previous study showed that professional isolation is associated with being valued at work among telecommuters [32]. Likewise, individuals with conventional professions may view their jobs as the backbone of society– and consequently feel less excluded. Furthermore, individuals with enterprising professions prefer to work with people, often influencing and persuading others (e.g., politicians, real estate agents, managers/CEOs). From their point of view, they could perceive their professions as being very well recognised. Combined with the presumably many (long-lasting) human contacts, this may explain why individuals with enterprising professions reported comparatively favorable outcomes across several sociodemographic groups. The marginal significant difference between realistic and investigative professions may be particularly explained by the fact that investigative professions (e.g., professors) often enjoy a very high social standing [33].

The fact that the differences are particularly present among individuals aged 65 and over may come as a surprise at first, since these individuals are often no longer working in their profession. At second glance, however, these results are conceivable, since older people presumably have more leisure time to reflect on their former professional environment (including in conversations with others). This recognition of the previous profession may also be retained during retirement. It is worth noting that our findings were also present– even after adjusting for economic factors (e.g., income or wealth).

Professional environment was mainly not associated with loneliness in our study. One former systematic review and meta-analysis showed that several job-related conditions (e.g., job performance, job satisfaction, worker-manager relationship, burnout) are associated with work-related loneliness [34]. Based on the findings from this previous work [34], one might assume that working conditions in particular could contribute to loneliness. If there are almost no differences in loneliness in our present study, one may assume that work-related conditions do not systematically differ between professional environments.

Sex-specific association between professional environments and perceived social isolation were identified in our study. This could be explained in particular by different perceptions of what society expects in terms of the professional environment for women and men. For example, women in artistic professions (such as graphic designers) and men in conventional professions (such as accountants) are more likely to fulfil (supposed) societal expectations. If such expectations are not met, individuals may feel excluded from society. However, the sample size for the artistic professions was quite small. These results should therefore be interpreted with great caution (and should not be over-interpreted). It is also worth noting that the effect sizes are rather small. This should be considered in particular with regard to its practical significance. Thus, further studies are strongly recommended.

This study has several noteworthy strengths and limitations. Notably, the data were derived from a large, nationally representative sample of individuals aged 43 and older living in the community. Sound tools were employed to quantify loneliness and perceived social isolation. A recently developed tool was used to quantify the professional environment based on Holland [14]. Even if the classification proposed by Holland [14] seems meaningful, there may be differences between the occupational groups within the six groups, which could be examined more closely in future research. Various covariates were incorporated into the regression model. One limitation is the cross-sectional nature of the data, which prevents us from ruling out that increases in loneliness or perceived social isolation could influence one’s professional environment. Nevertheless, in our view it is more plausible that the professional environment contributes to feelings of loneliness and perceived social isolation. Moreover, worth repeating that the sample size for some specific groups (artistic professions in particular) was rather small. This must be taken into account when interpreting the results.

In conclusion, our findings underlined the association between professional environment and perceived social isolation, with varying associations depending on sex and age. Enterprising professions in particular may assist in avoiding perceived social isolation, pending longitudinal evidence. Studies from other countries are strongly recommended.

## Electronic supplementary material

Below is the link to the electronic supplementary material.

Supplementary Material 1

## Data Availability

The data used in this study are third-party data. The anonymized data sets of the DEAS (1996, 2002, 2008, 2011, 2014, 2017, 2020, 2020/2021, 2023) are available for secondary analysis. The data has been made available to scientists at universities and research institutes exclusively for scientific purposes. The use of data is subject to written data protection agreements. Microdata of the German Ageing Survey (DEAS) are available free of charge to scientific researchers for non-profitable purposes. The FDZ-DZA provides access and support to scholars interested in using DEAS for their research. However, for reasons of data protection, signing a data distribution contract is required before data can be obtained. For further information on the data distribution contract, please see https://www.dza.de/en/research/fdz/access-to-data/application (accessed on 6 May 2025).
