# Supplementary material for: Association of professional environment with loneliness and perceived social isolation among individuals in their second half of life
Source: Aging Clin Exp Res. 2025 Jun 23;37(1):195. doi: 10.1007/s40520-025-03095-4 (PMC12185641; doi:10.1007/s40520-025-03095-4)
Supplement: Supplementary file 1 — Supplementary Material 1 [file 40520_2025_3095_MOESM1_ESM.docx]

Tukey-Kramer pairwise comparisons for variable professional environment (with loneliness as outcome; among the total sample)

mean

grp vs grp group means dif TK-test

-------------------------------------------------------

1 vs 2 1.8403 1.7281 0.1122 4.3497*

1 vs 3 1.8403 1.6905 0.1498 2.1611

1 vs 4 1.8403 1.6936 0.1467 8.6819*

1 vs 5 1.8403 1.7107 0.1296 6.9938*

1 vs 6 1.8403 1.7482 0.0920 5.2369*

2 vs 3 1.7281 1.6905 0.0376 0.5241

2 vs 4 1.7281 1.6936 0.0345 1.3813

2 vs 5 1.7281 1.7107 0.0174 0.6657

2 vs 6 1.7281 1.7482 0.0202 0.7934

3 vs 4 1.6905 1.6936 0.0031 0.0448

3 vs 5 1.6905 1.7107 0.0202 0.2910

3 vs 6 1.6905 1.7482 0.0578 0.8349

4 vs 5 1.6936 1.7107 0.0171 0.9860

4 vs 6 1.6936 1.7482 0.0547 3.3473

5 vs 6 1.7107 1.7482 0.0376 2.0846

Notes:

* < .05

Group 1 = realistic profession

Group 2 = investigative profession

Group 3 = artistic profession

Group 4 = social profession

Group 5 = enterprising profession

Group 6 = conventional profession

Tukey-Kramer pairwise comparisons for variable professional environment (with social isolation as outcome; among the total sample)

mean

grp vs grp group means dif TK-test

-------------------------------------------------------

1 vs 2 1.7380 1.5574 0.1806 6.7300*

1 vs 3 1.7380 1.6167 0.1213 1.7436

1 vs 4 1.7380 1.5805 0.1575 9.0023*

1 vs 5 1.7380 1.5592 0.1788 9.2921*

1 vs 6 1.7380 1.6084 0.1296 7.1237*

2 vs 3 1.5574 1.6167 0.0593 0.8211

2 vs 4 1.5574 1.5805 0.0231 0.8872

2 vs 5 1.5574 1.5592 0.0018 0.0674

2 vs 6 1.5574 1.6084 0.0510 1.9259

3 vs 4 1.6167 1.5805 0.0362 0.5229

3 vs 5 1.6167 1.5592 0.0574 0.8239

3 vs 6 1.6167 1.6084 0.0083 0.1196

4 vs 5 1.5805 1.5592 0.0212 1.1765

4 vs 6 1.5805 1.6084 0.0279 1.6488

5 vs 6 1.5592 1.6084 0.0491 2.6249

Notes:

* < .05

Group 1 = realistic profession

Group 2 = investigative profession

Group 3 = artistic profession

Group 4 = social profession

Group 5 = enterprising profession

Group 6 = conventional profession
